# Angiogenesis inhibitors in the treatment of prostate cancer

**DOI:** 10.1186/1756-8722-3-26

**Published:** 2010-08-02

**Authors:** Clara Hwang, Elisabeth I Heath

**Affiliations:** 1 Department of Internal Medicine, Henry Ford Hospital, CFP 559, 2799 West Grand Blvd, Detroit, MI 48202, USA; 2 Karmanos Cancer Institute and Wayne State University School of Medicine, 4234 KCI, 4100 John R, Detroit, MI, 48201, USA

## Abstract

Prostate cancer remains a significant public health problem, with limited therapeutic options in the setting of castrate-resistant metastatic disease. Angiogenesis inhibition is a relatively novel antineoplastic approach, which targets the reliance of tumor growth on the formation of new blood vessels. This strategy has been used successfully in other solid tumor types, with the FDA approval of anti-angiogenic agents in breast, lung, colon, brain, and kidney cancer. The application of anti-angiogenic therapy to prostate cancer is reviewed in this article, with attention to efficacy and toxicity results from several classes of anti-angiogenic agents. Ultimately, the fate of anti-angiogenic agents in prostate cancer rests on the eagerly anticipated results of several key phase III studies.

## Introduction

Prostate cancer, the second leading cause of cancer-related death in males, remains a major public health concern. Most cases of prostate cancer present with localized disease and may be cured with treatments such as surgery and radiation. However, as is true with most solid malignancies, the development of metastatic disease is ultimately lethal. Despite active systemic therapies, the metastatic phenotype is marked by the inevitable development of resistance, disease progression, and ultimately, death. Moreover, systemic treatments in prostate cancer are limited. Until recently, there were only three chemotherapeutic agents FDA-approved for use in castrate-resistant prostate cancer (estramustine, mitoxantrone, and docetaxel), with the most recent approval in 2004 [1-5]. Although 2010 is already notable for the approval of two additional agents for prostate cancer (sipuleucel-T and cabazitaxel) [1], there is still a clear need to develop additional systemic options in this deadly disease.

The observation of Dr. Judah Folkman that tumors are unable to grow more than 2-3 millimeters in the absence of neo-vascularization laid the foundation for the field of anti-angiogenic cancer therapy [6]. In addition, the observation that the process of angiogenesis could be stimulated by a diffusible substance released by tumor cells ultimately led to the identification of angiogenic factors which could be targeted for therapeutic use. After decades of active investigation, anti-angiogenic agents have finally reached the clinic. The first of these drugs to be FDA-approved is bevacizumab, which has now been approved for use in colon cancer, lung cancer, breast cancer, kidney cancer and glioblastoma [7-13]. To date, no anti-angiogenic agents have been approved for use in prostate cancer although clinical trials have suggested activity in this disease. The scope of this review is to provide an overview of molecular targets that are key components of angiogenic signaling and to discuss the results of anti-angiogenesis agents in prostate cancer clinical trials.

### Rationale for the use of angiogenesis inhibitors in cancer

Angiogenesis, or the process of new blood vessel formation, is necessary during cancer progression. Because growth of a tumor is dependent on the diffusion of nutrients and wastes, establishing a blood supply is critical for continued tumor enlargement. The limitation of nutrient diffusion is the reason why tumors are unable to grow larger than 2-3 mm in the absence of neovascularization. The transition of a tumor from this avascular state to acquiring the ability to promote the growth of new blood vessels has been termed the "angiogenic switch." This discrete change is a critical step in tumor progression.

Several processes have been described which compose the angiogenic switch [reviewed in [14]]. The endothelial cells that line existing blood vessels are activated, resulting in invasive, migratory, and proliferative properties. The basement membrane of the existing blood vessel and the surrounding extracellular matrix is degraded, allowing endothelial cell precursors to migrate toward the angiogenic stimulus. Endothelial cells proliferate and line the migration column. Capillary tubes are ultimately formed by the remodeling and re-adhesion of the endothelial cells, supported and stabilized by surrounding periendothelial cells and vascular smooth muscle cells.

The process of angiogenesis is stimulated by various angiogenic factors which are present in tumor and tumor-associated stroma. Although the most widely studied of these angiogenic factors is vascular endothelial growth factor-A (VEGF-A), the list of angiogenic activators includes other molecules such as placental growth factor, angiopoeitin-1, fibroblast growth factors, platelet-derived growth factor, epidermal growth factor and lysophosphatic acid. In addition, angiogenesis is inhibited by a number of naturally-occurring anti-angiogenic factors, which include thrombospondin-1, angiostatin, endostatin, tumstatin and canstatin. The balance of pro and anti-angiogenic factors is what ultimately determines the state of the angiogenic switch.

VEGF-A remains the best understood, and perhaps the most ubiquitous, of the pro-angiogenic growth factors [15]. As the name implies, members of the VEGF family act as growth factors, classically on vascular endothelial cells. VEGF-A is the prototypical member of the VEGF family of growth factors, which also includes placenta growth factor, VEGF-B, VEGF-C and VEGF-D. The VEGF family, in turn, is a sub-group of the platelet-derived growth factor family of cystine-knot growth factors. Members of the VEGF family act as ligands which bind to members of the VEGF receptor (VEGFR) family. There are three subtypes of the VEGFR family, and most of the known cellular responses appear to be mediated by VEGFR-2. VEGFR-3 appears to have a role in lymphangiogenesis; while VEGFR-1 may modulate VEGFR-2 signaling. In addition, VEGF ligands also bind to neuropilin receptors although the significance of this interaction is not as clearly understood. When VEGF ligand binds to VEGFR, downstream signaling is mediated through dimerization of the receptor and subsequent phosphorylation of receptor tyrosine residues. This activation results in multiple downstream signals that ultimately drive the angiogenesis process. The cellular effects of VEGF-A when bound to VEGFR-2 on endothelial cells include vasodilatation, vascular permeability, mitogenesis, invasive properties and chemotaxis.

VEGF-A is produced both by tumor cells as well as tumor-associated stromal cells [16], with VEGF-A expression most clearly induced by hypoxic conditions. Cells respond to hypoxic conditions through the modulation of hypoxia-inducible factors (HIFs). HIF-1 is a highly evolutionarily conserved member of the basic-helix-loop-helix family of transcription factors [17]. HIF-1 is a heterodimer that contains an alpha and a beta subunit (HIF-1α and HIF-β). HIF-1α is hydroxylated by HIF prolyl-hydroxylase, which then targets HIF-1α for degradation under normoxic conditions. Hydroxylated HIF-1α is specifically ubiquitinated by the VHL E3 ubiquitin ligase, marking HIF-1α for proteasomal degradation. Under hypoxic conditions, the hydroxylation of HIF-1α is limited by the availability of oxygen molecules and HIF-1α is stabilized and accumulates. HIF-1α can then dimerize with HIF-β and induce the transcription of hypoxia-survival genes. Among the transcripts regulated by HIF-1 is VEGF, which allows tissues to adapt to hypoxic conditions by promoting angiogenesis.

Although VEGF signaling has been the most closely associated with tumor angiogenesis, special mention will also be made here regarding PDGF pathways, because of the availability of clinical agents that modify PDGF signaling. Similar to VEGF, members of the PDGF family of growth factors dimerize and interact with members of the PDGF-R family of tyrosine kinase receptors. PDGF signaling has been implicated in tumorigenesis through several mechanisms, including proliferative autocrine signaling, promotion of invasive and metastatic behaviors through control of the epithelial-mesenchymal transition, and paracrine recruitment of stromal cells, including effects on angiogenesis [reviewed in [18]]. As a result of these pleotropic effects, PDGF-targeting agents are being investigated for their potential as anti-neoplastic therapy [19].

Understanding the mechanisms behind angiogenesis has led to the availability of novel drugs that target components of the angiogenesis pathway that are now being utilized in cancer therapy. The advent of an entirely new class of anti-cancer therapies has required an understanding of the differences between angiogenesis inhibitors and more conventional chemotherapeutic agents. The use of angiogenesis inhibitors has been postulated to have some theoretical advantages and disadvantages over traditional chemotherapy. Because most tissues in a mature organism do not rely on angiogenesis, angiogenesis inhibition may have a greater therapeutic index than cytotoxic agents, which are also toxic to many normal cells. This hypothesis has been shown to be at least partially true when angiogenesis inhibitors have been studied in clinical trials; investigators have found that angiogenesis inhibitors have a toxicity profile that is generally favorable to cytotoxic agents with the notable exception of unique vascular toxicities.

In addition, it has been argued that because endothelial cells do not possess the genetic instability of cancer cells, resistance may be less of an issue with anti-angiogenesis therapy. As our knowledge and experience has increased, it has become clear that this was likely an overly naïve characterization of anti-angiogenic therapy. Various mechanisms of resistance to angiogenesis therapy have been outlined [reviewed in [15,20,21]]. Because of redundancy in angiogenic signals, angiogenesis inhibition using a single target can be overcome by shifting the balance of other pro- and anti-angiogenic signals. For example, if signal transduction through the VEGF receptor is targeted, resistance could develop by tumor overexpression of VEGF. If VEGF is targeted, tumors may secrete a different pro-angiogenic factor. Since tumors play a central role in the angiogenic signaling pathways, the genetic instability of the tumor will contribute to angiogenesis-inhibitor resistance. Clonal evolution and tumor adaptation may also result in a tumor that is tolerant of hypoxic conditions and subsequently less dependent on neovascularization. In addition, it has been proposed that hypoxia may select for, or even induce, clones with greater invasive and metastatic potential. Acquired tumor resistance may be a result of evolutionary, genetic, hypoxic or physiologic changes in tumor biology. Changes in expression of angiogenic factors by stromal cells are now also felt to be a key factor in mediating angiogenesis-resistance. These stromal changes may be mediated by a physiologic response to hypoxia, by tumor-recruitment of stromal cells, tumor-secretion of stromal-stimulating factors, or other mechanisms.

One final difference between angiogenesis inhibitors and cytotoxic therapies that has proven to be critical in designing and interpreting clinical trials is that angiogenesis inhibition may arrest tumor growth in a dormant state without tumor regression, because the tumor cells are not directly targeted. The first implication of this fact is that traditional endpoints, such as radiographic criteria for measuring response, may not be an accurate measure of anti-tumor efficacy. In addition, it has been shown that tumors held in a dormant state by angiogenesis inhibition can grow vigorously if the inhibition is released. Thus, there may be a greater role for maintenance therapy when using angiogenesis inhibitors. In addition, the question of whether to continue an anti-angiogenic agent in the face of disease progression remains an open question.

### Evidence for the role of angiogenesis in prostate cancer pathogenesis

In addition to the evidence that angiogenesis may be important for tumor growth in general, there is a growing body of evidence that angiogenesis plays a role in prostate cancer in particular. It has been demonstrated that prostate cancer cells express VEGF [22,23] and that the expression of VEGF by neoplastic cells is greater than that found in normal prostate stromal tissue. Moreover, blood and urine VEGF levels have been shown to correlate with prostate cancer patient outcomes [24-26]. Markers of neovascularization have also been shown to have significance in prostate cancer. Microvessel density has been used as a surrogate histologic measure of angiogenesis within a tumor. Microvessel density in prostate cancer has been shown to correlate strongly with Gleason grade and predicts disease progression [27,28]. As yet, it has not been shown definitively that microvessel density can be used as an independent predictor of patient outcome. Also, whether neovascularization is the primary pathogenic event, or whether simply a reflection of the hypoxic conditions that result from unchecked growth, is unclear from these histologic correlations. However, this observation does provide a rationale for further exploring the role of angiogenesis in prostate cancer progression.

Preclinical data have provided some evidence that anti-angiogenic therapy is more effective in the setting of minimal tumor burden. This concept was demonstrated in a prostate cancer mouse model where VEGFR antagonists only inhibited tumor progression before tumors produced significant levels of VEGF [29]. Prostate cancer offers a unique clinical scenario to test the hypothesis that angiogenesis-inhibition will be more effective in the setting of minimal disease, because in the PSA era, disease recurrence is often detected before metastatic deposits are detectable by imaging modalities or physical examination.

### Clinical trials with anti-angiogenic agents in prostate cancer

#### Bevacizumab - VEGF-targeting monoclonal antibody

Bevacizumab is a humanized monoclonal antibody that recognizes all VEGF isoforms, preventing binding to the VEGF receptor. It was developed from a murine anti-human VEGF antibody and retains 7% of the murine sequence. Single agent bevacizumab was initially evaluated in 15 patients with castrate-resistant cancer [30]. Bevacizumab was given at a dosage of 10 mg/kg every two weeks for six treatments. Treatment was continued for patients with either response or stable disease. There were no patients who had a PSA decline of more than 50%, although four patients out of fifteen had PSA declines of less than 50%. There were no objective responses at day 70. The study was thus interpreted as a negative study and highlights some of the difficulties in designing and interpreting the results of clinical trials with anti-angiogenic agents. For anti-angiogenic agents that are more likely to be cytostatic and not cytotoxic, radiographic and PSA rates may not be the best measure of clinical activity. The authors also suggested that evaluating the activity of angiogenesis inhibitors in earlier stages of disease may yield more promising results. Interestingly, Iacobelli presented a case report of a patient with castrate-resistant prostate cancer who was treated with single agent bevacizumab when he refused chemotherapy [31]. Bevacizumab 7.5 mg/kg every 14 days was used for more than six months with reduction in PSA from 14 to 4 ng/ml in one month as well as radiographic response of retroperitoneal lymphadenopathy. Single agent bevacizumab in prostate cancer is currently being evaluated in patients with biochemical recurrence to assess the hypothesis that single agent bevacizumab may have activity in patients with a lesser burden of disease. In addition to PSA declines of at least 50%, time to PSA progression is a primary outcome measure of this study. Change in PSA velocity is a secondary outcome measure, which may better measure the cytostatic effects of bevacizumab.

Bevacizumab has also been studied in combination with cytotoxic agents in prostate cancer. A cooperative group study, CALGB 90006, used bevacizumab in combination with docetaxel and estramustine [32,33] in prostate cancer patients who were chemotherapy-naïve. Docetaxel was given at 70 mg/m2 every three weeks in combination with estramustine 280 mg three times daily on days one through five. Bevacizumab was given at 15 mg/kg on day 2 of the chemotherapy cycle. 79 patients were enrolled. Although final results have not been published, the study reported a PSA decline of more than 50% in 77% of patients [33]. 42% of patients with measurable disease were noted to have partial response based on measurable disease. Bevacizumab was also given in combination with docetaxel in a phase II study of 20 patients with docetaxel-refractory metastatic prostate cancer [34]. All patients had been previously treated with both docetaxel and mitoxantrone; many had been treated with third-line chemotherapy or beyond. Patients were treated with docetaxel 60 mg/m2 and bevacizumab 10 mg/kg every three weeks. PSA declines of ≥ 50% were seen in 55% of patients. Objective radiographic response was seen in three of 8 patients with measurable disease. In addition, PSA declines of ≥ 50% and radiographic responses were observed in patients who did not respond to initial docetaxel chemotherapy.

From these phase II studies, it was concluded that the combination of bevacizumab and chemotherapy is reasonably safe and has activity in prostate cancer. The activity of these bevacizumab regimens compared favorably to historical controls [4,5]. However, because these were phase II studies, it could not be determined whether the addition of the anti-angiogenesis agent contributed significantly to the clinical benefit of the regimen, since chemotherapy alone also has activity in prostate cancer and comparison to historical controls cannot be considered conclusive evidence of benefit. This question was addressed by a randomized phase III (CALGB 90401) comparing docetaxel and prednisone to the combination of docetaxel, prednisone, and bevacizumab in patients who are chemotherapy-naïve. Enrollment on this clinical trial was completed in early 2008 and results were recently reported in abstract form [35]. This study randomized 1050 patients with chemotherapy-naïve, metastatic castrate-resistant prostate cancer (mCRPC) to receive docetaxel plus prednisone (docetaxel 75 mg/m2 on day 1; prednisone 5 mg po BID) with either bevacizumab 15 mg/kg or placebo given on day 1 of a 21-day cycle. The study did not meet its primary endpoint of overall survival, and the bevacizumab arm was notable for a higher rate of both treatment-related toxicity and mortality. The rate of grade 3 adverse events in the bevacizumab arm was 74.8% compared to 55.3% in the placebo arm (p < 0.001). In addition, there was a 4.4% toxic death rate on the bevacizumab arm, compared to a rate of 1.1% in the placebo arm (p = 0.0014). A majority of the treatment-related deaths were related to infection. However, it is important to point out that the bevacizumab arm was superior in several measures of anti-cancer efficacy: progression-free survival, rates of ≥ 50% PSA decline, and objective response rate. The median progression-free survival was 9.9 months on the experimental bevacizumab arm and 7.5 months on the control arm (p < 0.0001). The PSA response rate was 69.5% in the experimental arm, compared to 57.9% on the control arm, with a p value of 0.0002. Finally, there was also a statistically significant benefit of bevacizumab in the measurable disease response rate (53.2% vs. 42.1%, p = 0.0113).

Although there was a trend for an improvement in overall survival in the bevacizumab arm (22.6 vs. 21.5 months, p = 0.181), this difference was not statistically significant. Several reasons have been suggested to explain the discordance between the overall survival and progression-free survival endpoints. To begin, the median overall survival in the control arm was 21.5 months, which is longer than previously reported studies. The selection of healthier patients, perhaps earlier in their disease course, has been attributed to patient and physician enthusiasm. The improved overall survival in the control arm may have limited the ability to detect a treatment effect because of reduced statistical power. In addition, preliminary subset analysis suggested a more pronounced overall survival effect in patients with poorer prognosis (lower hemoglobin, higher alkaline phosphatase, elevated LDH). Thus, the selection of generally healthier patients may have masked any effect of bevacizumab on overall survival, in addition to limiting the statistical power of the study. Secondly, duration of therapy has been cited as an important contributor to treatment-efficacy, especially with anti-angiogenic therapy. The median number of treatment cycles was only 8 cycles, compared to 9.5 cycles on the TAX 327 study [4]. Because the trial was designed prior to consensus recommendations discouraging treatment-discontinuation for isolated PSA progression in the absence of clinical progression, it is possible that bevacizumab was discontinued prematurely on the basis of PSA progression alone. The effect of premature discontinuation would be compounded by the postulated aggressive "rebound" phenomenon that has been reported to occur upon the discontinuation of anti-angiogenic therapies. Finally, the availability of subsequent therapies, as well as the excess toxicity in the bevacizumab arm may also have mitigated the effect of bevacizumab on overall survival in this population.

### VEGFR tyrosine kinase inhibitors

As previously mentioned, VEGF ligands stimulate angiogenesis by binding and activating VEGF tyrosine kinase receptors. The development of small molecules that inhibit tyrosine kinases, primarily by binding the ATP binding domain, has led to investigation of tyrosine kinase inhibitors as angiogenesis inhibitors. In general, because tyrosine kinases are involved in many signaling pathways and the ATP binding sites are relatively conserved, tyrosine kinase inhibitors may target more than one receptor pathway that has a role in tumor progression.

#### Sorafenib

Several tyrosine kinase inhibitors are known to target the VEGF tyrosine kinase receptors. Sorafenib is a multikinase inhibitor that can target tumor cell proliferation by Raf kinase inhibition, in addition to targeting angiogenesis by inhibiting the VEGFR-2, VEGFR-3 and PDGFR kinases. Results of three phase II studies in prostate cancer have been recently reported. Twenty-two patients with metastatic androgen independent prostate cancer were enrolled onto a Phase II NCI-sponsored study of sorafenib 400 mg twice daily [36]. A majority of patients (59%) had received at least one chemotherapy regimen prior to enrolling on this study. No complete or partial responses were seen. There were no patients with PSA decline ≥ 50%. Although these measures of disease activity were negative, PSA declines were seen after discontinuation of study agent in the absence of starting any new therapy. In addition, two patients with rising PSA levels showed resolution of bone lesions on bone scan. The authors presented data that sorafenib can result in PSA secretion in vitro, potentially explaining these results.

The Canadian experience with sorafenib as a single agent in prostate cancer is similar to the other Phase II studies [37]. Twenty-eight patients were treated with sorafenib 400 mg twice daily. PSA progression despite castrate levels of testosterone was required for eligibility and prior chemotherapy was not permitted. All but two patients had evidence of metastatic disease. No patient had radiographic response using RECIST criteria. Only one patient (3.6%) had a PSA decline of ≥ 50%, from 10,000 to 1643. However, 10 of 16 patients who did not receive any post-sorafenib treatment had subsequent PSA declines. In combination with the previous data from Dahut et al [36], these clinical observations imply that PSA progression may not be the best reflection of disease activity in the setting of sorafenib treatment.

Finally, a European study also reported their Phase II results with sorafenib as a single agent used at a dose of 400 mg twice daily [38]. In contrast to the NCI study, the 55 men enrolled on the trial all had chemotherapy-naïve castrate-resistant prostate cancer. No responses were seen by RECIST criteria in eight patients with measurable disease. Two patients had ≥ 50% PSA declines at 12 weeks. Taken together, sorafenib shows little clinical activity in prostate cancer as a single agent, although intriguing evidence regarding PSA increases due directly to sorafenib may underestimate its effects on PSA progression. Sorafenib is currently being studied in earlier stages of disease, as well as in combination with chemotherapy.

#### Sunitinib

Sunitinib is another multitargeting tyrosine kinase inhibitor that inhibits VEGF and PDGF receptors, among others. A case report of a male whose prostate cancer was being managed by active surveillance while he was treated with sunitinib for metastatic renal cell carcinoma showed evidence of both PSA decline ≥ 50% as well as radiographic and histologic evidence of regression [39]. A more formal phase II study was recently reported by Michaelson et al [40]. Thirty-four men, half chemotherapy-naïve and half docetaxel-resistant, were treated with sunitinib 50 mg daily for four weeks followed by two weeks rest. All but one patient had metastatic disease. Sunitinib did not appear to have significant activity when measured by classic criteria. Only two patients had PSA decline ≥ 50%; and the best radiographic response was partial response in one patient. Eighteen men had stable disease at twelve weeks by RECIST criteria. As seen in the sorafenib studies, PSA decline did not correlate with radiographic imaging. The sole patient with partial response by RECIST criteria had a PSA decline of less than 50%. In addition, patients with radiographic improvement that did not meet RECIST criteria for response were noted to have rising PSA levels. Thus, the activity profile of sunitinib appears to be similar to that of sorafenib in this setting. The findings of this study again highlight the need for better markers of clinical benefit in anti-angiogenesis strategies.

### Thalidomide

Thalidomide was originally marketed as an oral sedative and anti-emetic drug in the 1950's. However, it was subsequently withdrawn from the market because of reports of teratogenicity, including phocomelia and other limb deformities. Subsequent work suggested that thalidomide had anti-angiogenic properties that may be responsible for its teratogenic effects [41]. Confirming this hypothesis, thalidomide given to prostate cancer patients prior to surgery resulted in reduced microvessel density as well as decreased expression of VEGF and IL-6 in prostatectomy specimens [42]. Thalidomide also appeared to affect other components of the tumor microenvironment without affecting the epithelial component itself. Sonic hedgehog signaling and the ratio of matrix metalloproteinases to E-cadherin were both reduced, suggesting a less aggressive molecular phenotype. The underlying mechanism of angiogenesis inhibition by thalidomide, as well as its other biologic activities, is still not entirely understood.

Motivated by the discovery of its anti-angiogenic effects, thalidomide was studied as a single agent in castrate-resistant prostate cancer [43]. Two dose levels were planned, but because of tolerability, the majority of patients were treated at the low dose of 200 mg/day. A majority of the 63 patients enrolled had metastatic disease, with a median PSA of 326 ng/mL. 24% of patients had received previous chemotherapeutic agents. Response rates to thalidomide were not dramatic, but thalidomide did show some evidence of activity in this cohort of patients. Nine patients (14%) had a PSA decline of ≥ 50% and 17 patients (27%) had at least a PSA decline of 40%. Because thalidomide was shown to increase PSA secretion in vitro [44], PSA declines of less than 50% were felt to be important to report. One patient had a PSA decline of ≥ 50% that lasted for more than one year. No objective radiographic responses were observed.

A randomized Phase III study of thalidomide in patients with biochemically recurrent, castrate-sensitive disease treated with intermittent androgen deprivation was recently reported [45]. 159 patients were enrolled and were treated with six months of GnRH agonist therapy followed by thalidomide 200 mg daily or placebo. At the time of progression, patients were restarted on six months of androgen deprivation and crossed over to the alternate drug. During both phases of therapy, time to PSA progression favored the thalidomide group (15 vs. 9.6 months in the first phase; 17.1 vs. 6.6 months in the second phase). The difference between the groups during the second, cross-over, phase was statistically significant (p = 0.0002), while the difference in the first phase of treatment was not statistically significant. The application of these findings to clinical practice is limited by the unclear relationship between PSA progression and clinical benefit, especially during the treatment of castrate-sensitive prostate cancer with intermittent androgen deprivation.

Thalidomide has also been tested in combination with chemotherapy in several phase II studies. In a randomized phase II study, 75 patients with metastatic castrate resistant prostate cancer received weekly docetaxel, 30 mg/m2 for three weeks of a four week cycle with or without thalidomide 200 mg daily [46-48]. In the initial report of the fully accrued trial, the percentage of patients with PSA declines ≥ 50% was greater in the combination arm (53% vs. 37%). Median overall survival was reported as 14.7 months for docetaxel monotherapy and 28.9 months in the combination arm. These differences were not statistically significant when initially reported; however, updated results demonstrated an overall survival of 25.9 months on the thalidomide arm and 14.7 months for the docetaxel monotherapy arm (p = 0.0407) [48]. A high rate of thromboembolic complications occurred (12 of initial 43 patients on combination arm) and thromboprophylaxis was subsequently recommended. Figg et al also reported on the results of a phase II study of 20 patients treated with weekly docetaxel, thalidomide 200 mg daily, and estramustine [49]. The patient population had metastatic disease that was androgen-insensitive but chemotherapy-naïve. 90% demonstrated ≥ 50% PSA declines; two of 10 patients with measurable disease had a partial response; and time to progression was 7.2 months.

The activity of thalidomide, bevacizumab, and docetaxel in 60 chemotherapy-naïve patients with metastatic castrate-resistant prostate cancer was reported by Ning et al [50]. Patients were given docetaxel at 75 mg/m2 every 21 days; bevacizumab 15 mg/kg every 21 days; thalidomide 200 mg daily; prednisone 5 mg twice daily; and thromboprophylaxis with enoxaparin. 90% of patients had PSA decline of ≥50% and progression free survival was estimated at 18.2 months. Median overall survival was reported as 28.2 months. Although the activity also compares favorably to the original TAX 327 data (18.9 month OS and 45% of patients with PSA declines of ≥ 50% [4]), the comparison to historical controls suffers from the usual limitations. In addition, thalidomide toxicity required dose reduction in many patients.

While there is suggestion of thalidomide activity in both castrate-resistant and castrate-sensitive prostate cancer, further phase III studies are needed to clarify its role in prostate cancer therapy. In addition, follow-up of the phase III thalidomide study in combination with intermittent androgen deprivation may be revealing to see if the differences seen in the time to PSA progression will ultimately result in differences in clinical endpoints such as metastatic disease progression or overall survival; however, the cross-over design may complicate analysis of longer-term endpoints. Notably, lenalidomide, a thalidomide derivative with a more favorable toxicity profile, is also being studied in prostate cancer. Preliminary phase I-II results as a single agent have been reported in abstract form [51]. Lenalidomide is also being evaluated in combination with both chemotherapy and other anti-angiogenesis agents. Given previous results discussed with a thalidomide, bevacizumab, docetaxel combination [50], the NCI is sponsoring a phase II study to evaluate toxicity and efficacy of the less-toxic lenalidomide, in combination with bevacizumab, docetaxel and prednisone (NCT00942578). Finally, a phase III study of the combination of docetaxel with and without lenalidomide is currently underway (NCT00988208). A summary of the clinical trials investigating VEGF-targeting therapies and thalidomide-derivatives in prostate cancer is presented in Table 1.

**Table 1:** A summary of clinical trials with angiogenesis inhibitors in prostate cancer.

Drug(s)		N	Population	Clinical benefit	Ref
**VEGF monoclonal antibody**					
Bevacizumab 10 mg/kg q2wk × 6	Ph II	15	mCRPC	4 of 15 had PSA decline < 50% No PSA decline > 50% No objective responses	[30]
Bevacizumab 15 mg/kg d2 Docetaxel 70 mg/m2 q3wk Estramustine 280 mg TID d1-5	PhII	79	mCRPC	PSA response > 50% in 77% of patients 42% with radiographic partial response	[32,33]
Bevacizumab 10 mg/kg q3wk Docetaxel 60 mg/m2	PhII	20	mCRPC, docetaxel failure	PSA response > 50% in 55% of patients 3 of 8 patients had objective radiographic response	[34]

**Tyrosine Kinase Inhibitor**					
Sorafenib 400 mg BID	PhII	22	mCRPC,	No PSA decline > 50% No objective radiographic responses	[36]
Sorafenib 400 mg BID	PhII	28	CRPC, docetaxel-naïve	PSA response > 50% in 1 patient (3.6%) No objective radiographic responses	[37]
Sorafenib 400 mg BID	PhII	55	CRPC, docetaxel-naïve	PSA response > 50% in 2 patients (3.6%) No objective radiographic responses	[38]
Sunitinib 50 mg/day × 4 wks of 6 wk cycle	PhII	34	CRPC	PSA response > 50% in 2 patients (5.9%) 1 objective radiographic response (2.9%)	[40]

**Thalidomide**					
Thalidomide 200 mg/day	PhII	63	CRPC	PSA response > 50% in 14% of patients No objective radiographic responses	[43]
Thalidomide 200 mg/day	PhIII	159	bCSPC	Crossover design, time to restarting intermittent ADT Time to PSA progression favored thalidomide group 15 v 9.6 mo, p = 0.21 in first phase 17.1 v 6.6 mo, p = 0002 in second phase	[45]
Thalidomide 200 mg/day Docetaxel 30 mg/m2 d1, 8, 15 of 28 day cycle	rPhII	75	mCRPC, docetaxel-naïve	PSA response > 50% in 53% of thalidomide group vs PSA response > 50% in 37% of control group (p = 0.32) OS of 25.9 mo in thalidomide group vs OS of 14.7 mo in control group (p = 0.0407)	[46-48]
Thalidomide 200 mg/day Docetaxel 30 mg/m2 d1, 8, 15 Estramustine TID d1-3, 8-10, 15-17 of 28 day cycle	PhII	20	mCRPC, docetaxel-naïve	PSA response > 50% in 90% of patients	[49]
Thalidomide 200 mg/day Docetaxel 75 mg/m2 q3wk Bevacizumab 15 mg/kg q3wk	PhII	60	mCRPC, docetaxel-naïve	PSA response > 50% in 88% of patients	[50]

**Pending Phase III studies**					
Docetaxel + Prednisone +/- Bevacizumab	PhIII	1050	mCRPC, docetaxel-naïve	Preliminary results indicate no benefit in overall survival for bevacizumab arm	[35]
Docetaxel + Prednisone +/- Lenalidomide	PhIII	1015*	mCRPC, docetaxel-naïve	Results pending	NCT00988208
Prednisone +/- Sunitinib	PhIII	819*	mCRPC, docetaxel failure	Results pending	NCT00676650

*Anticipated Enrollment. mCRPC - metastatic castrate-resistant Prostate Cancer. bCRPC - biochemically recurrent castrate-resistant prostate cancer. bCSPC - biochemically recurrent castrate-sensitive prostate cancer.

### PDGF-targeted therapy

As mentioned above, PDGF has angiogenic properties. In addition to the effects of PDGF on angiogenesis, there is other evidence suggesting a role for PDGF-targeted therapy in the treatment of prostate cancer. PDGFR was seen as the most commonly amplified transcript when aspirates from prostate cancer bone metastases were evaluated for amplification of tyrosine kinase receptors, and overexpression of PDGF in prostate cancer bone metastases was confirmed by immunohistochemistry [52]. PDGF inhibitors have also been shown to reduce interstitial fluid pressure in tumors, enhancing delivery of chemotherapy to tumors [53]. Unfortunately, clinical trials using PDGF-targeting therapy in patients with prostate cancer have been disappointing.

Imatinib is a multi-tyrosine kinase inhibitor with anti-PDGFR activity. It is used clinically in the setting of chronic myelogenous leukemia and GI stromal tumors, where inhibition of the bcr-abl and c-kit tyrosine kinase receptors has significant clinical effects. Imatinib has been used as a single agent in three phase II studies in the setting of biochemically relapsed prostate cancer [54-56]. Lin et al studied imatinib at a dose of 400 mg orally twice daily in 20 patients with nonmetastatic prostate cancer and rising PSA. Only one patient had PSA decline of ≥ 50%. Overall, there was no significant change in PSA doubling time after imatinib treatment. In addition, 11 men withdrew from the study because of toxicity. The trial was stopped early because grade 3-4 toxicity events were higher than the predetermined target of 5%. Rao et al also reported results of a phase II study using imatinib 400 mg orally twice daily in 21 patients with PSA-only recurrence. This trial was stopped early because five patients were noted to have unusually fast PSA rise while on study. Toxicity was also moderate, with six patients withdrawing consent for toxicity. No patient was seen to have a PSA decline of ≥ 50%. Bajaj et al also reported their results using imatinib 400 mg orally twice daily in a similar patient population. PSA declines of ≥ 50% were seen in only two of 27 patients (3.7%), with the majority demonstrating PSA progression (74.1%). In addition, toxicity was not infrequent, with grade 3 toxicities seen in approximately 20% of patients. Seven patients withdrew from the study for toxicity. Taken together, these three phase II studies demonstrate that imatinib 400 mg twice daily has little effect on PSA kinetics and is too toxic to consider as therapy in biochemical recurrence, which is typically an asymptomatic population.

The effect of PDGF-targeting has also been evaluated in the metastatic setting. The effect of an intravenous PDGFR inhibitor, SU101 (leflunomide) in men with androgen-independent prostate cancer was assessed in a phase II study that enrolled 44 men [57]. All patients had metastatic disease and half the patients had received previous chemotherapy. SU101 was given intravenously with a 4 day loading dose followed by weekly infusions (all but one patient received a dose of 400 mg/m2/day). Three patients evaluable for PSA response had PSA decline of ≥ 50%. One of these patients had a dramatic decline from 293 to 0.3 ng/mL. This same patient was noted to have an objective partial response, out of 19 patients with measurable disease. Although the clinical results were not encouraging, the observation of an objective response with SU101 therapy suggests the possibility that there may be a small subset of prostate cancer that will benefit from PDGF signaling inhibition.

Finally, imatinib has been combined with docetaxel in a randomized phase II study in men with metastatic androgen-independent prostate cancer [58]. 144 patients were enrolled and randomized to receive either imatinib 600 mg daily or placebo. In addition, patients received docetaxel 30 mg intravenously on days 1, 8, 15, and 22 of a 42 day cycle. Most men were chemotherapy-naïve (approximately 70% in both groups). The PSA response rate (declines ≥ 50%), progression-free survival, and overall survival were not significantly different in the imatinib group, and in fact, generally favored the placebo arm. The trial was stopped early because of toxicity concerns, with gastrointestinal toxicities predominating.

### Other approaches

In addition to the agents discussed above, other angiogenesis inhibitors are actively being evaluated in prostate cancer. Aflibercept, also called VEGF-trap, is a fusion protein that combines the Fc portion of human IgG1 with the VEGFR-1 and -2 ligand binding domains. Aflibercept binds VEGF-A, VEGF-B and Placental-GF, competitively inhibiting VEGF receptor activation. The VENICE study is currently enrolling 1200 patients with castrate-resistant prostate cancer in a Phase III evaluation of docetaxel/prednisone with and without aflibercept to definitively answer the question whether this drug has an additive benefit to docetaxel-based chemotherapy in prostate cancer. Other tyrosine kinase inhibitors are also being considered for use in prostate cancer. For example, AZD2171, also known as cediranib, is an oral tyrosine kinase inhibitor that potently targets VEGFR-1, -2 and -3 while also having lesser effects on PDGFR and c-kit [59]. This drug is currently being studied in metastatic castrate-resistant prostate cancer in Phase II clinical trials. Preliminary reports of responding prostate cancer patients on phase I and phase II studies [60,61], suggested that PSA response did not correlate well with partial responses seen on imaging, reminiscent of similar experiences with sorafenib. In addition, the tyrosine kinase inhibitor pazopanib, which targets VEGF and PDGF receptors [62], is currently undergoing clinical investigation in prostate cancer in the castrate-resistant setting (NCT00454571, NCT00486642, NCT00945477).

Another agent with anti-angiogenic properties that is being evaluated in clinical trials is tasquinimod. This compound was initially identified on the observation that linomide, an agent being investigated in multiple sclerosis, had anti-angiogenic properties [63]. Since the original compound was found to be toxic in clinical trials, analogs were screened for anti-angiogenic activity. Although its mechanism of anti-angiogenic activity is not entirely clear, tasquinimod was identified as a lead compound. Subsequently, it was shown that tasquinimod has anti-tumor activity in prostate cancer xenograft models and was well-tolerated in phase I studies [63,64]. Most recently, results of a randomized phase II study were reported in abstract form [65]. 206 patients with asymptomatic, metastatic CRPC were assigned in a 2:1 ratio to either oral tasquinimod or placebo. Tasquinimod met the primary endpoint of the study, which was a superior progression-free proportion at six months compared to placebo (69% vs. 34%, p < 0.0001). In addition, median progression-free survival also favored tasquinimod (7.6 vs. 3.2 months, p = 0.0009). Progression was defined only clinically, without the use of PSA criteria. Notably, tasquinimod had no appreciable effect on PSA compared to placebo. Toxicity included on-target toxicity such as vascular events, but was felt to be manageable by investigators. Overall, the results of this trial were felt to justify the planning of a phase III study.

In addition to the approaches just considered, review of known angiogenesis mechanisms suggests other ways to target this process for clinical benefit. In fact, other anti-angiogenesis approaches are being pursued in cancer, although they are not as mature in their application for prostate cancer. For example, as discussed above, the angiogenic switch is triggered by a balance of pro- and anti-angiogenic factors. We have discussed in great detail a single pro-angiogenic factor, the VEGF family. However, there are naturally occurring anti-angiogenesis factors which exist, such as endostatin and thrombospondin. Compounds which mimic the action of these natural anti-angiogenic factors are also being evaluated for use in solid malignancies [66,67]. In addition, the production of pro-angiogenesis factors is also being targeted with HIF-1α inhibitors [68].

As it has become clearer that resistance to angiogenesis inhibition can present a clinical challenge, targeting the process from multiple angles may provide synergy or additive effects able to overcome resistance. Results of the combination of docetaxel, bevacizumab and thalidomide in prostate cancer are encouraging, as discussed above [50]. Dual inhibition is also being investigated in a phase I study with the combination of sorafenib and bevacizumab (NCT00098592). However, this approach of dual targeting will require proceeding with caution to avoid unexpected toxicities. A phase I strategy of dual inhibition using sunitinib and bevacizumab in renal cell carcinoma was complicated by the development of frequent severe hypertension and microangiopathic hemolytic anemia associated with reversible posterior leukoencephalopathy syndrome [69]. Although microangiopathic hemolytic anemia and reversible posterior leukoencephalopathy syndrome were not reproduced in an independent phase I combination study performed in all tumor types, toxicity-related dose modifications were frequently necessary [70]. Various strategies have been proposed to mitigate the toxicity of anti-angiogenic combinations. These include monitoring pharmacodynamic endpoints instead of escalating to maximally tolerated dose of each agent, or limiting exposure to a drug by restricting its administration to a short pulse at a critical point in the chemotherapy cycle.

## Conclusion

Angiogenesis appears to play a role in the progression of prostate cancer. After several decades of investigation, angiogenesis inhibitor therapy is finally being evaluated in prostate cancer patients. While anti-angiogenic agents appear to be a promising addition to prostate cancer therapies, challenges in clinical trial design and interpretation have prevented the rapid adoption of these agents into clinical practice. Among these challenges is the fact that certain anti-angiogenic agents can increase PSA in the face of evidence of disease response. To address this concern, the 2008 Prostate Cancer Clinical Trials Working Group (PCWG-2) has recommended specific endpoints for cytostatic therapies, including anti-angiogenesis agents, which emphasize time-to-event endpoints [71]. Moreover, PCWG-2 stresses the importance of radiographic and symptomatic progression when making clinical trial treatment decisions and discourages investigators from discontinuing treatment on the basis of isolated PSA progression. Attention to such clinical endpoints may limit premature discontinuation of therapy, which has been cited as a contributor to the negative results of CALGB 90401. Future clinical development of anti-angiogenic therapy will benefit from attention to these design considerations. So far, evidence that the use of angiogenesis inhibitors results in meaningful clinical benefit for prostate cancer remains elusive, including recent data from CALGB 90401. Nonetheless, continued enthusiasm for anti-angiogenesis therapies in prostate cancer has been justified by signs of activity on CALGB 90401, as well as encouraging phase II data, including the combination of bevacizumab and thalidomide with docetaxel [50]. Final results from several Phase III studies in the castrate-sensitive, docetaxel-naïve, and docetaxel-refractory settings are still pending. Results from these clinical trials will hopefully clarify the role of angiogenesis inhibitors in the arsenal of prostate cancer therapies.

## List of Abbreviations

CRPC, Castrate-resistant prostate cancer; ATP, Adenosine triphosphate; bCRPC,  biochemically-recurrent castrate-resistant prostate cancer; bCSPC, biochemically-recurrent  castrate-sensitive prostate cancer; CALGB, Cancer and Leukemia Group B;  FDA, Food and Drug Administration; GnRH, Gonadotropin Releasing Hormone; HIF,  Hypoxia-inducible factor; mCRPC, metastatic Castrate-resistant prostate cancer; NCI,  National Cancer Institute; PDGF, Platelet-derived growth factor; PDGFR, Platelet-derived  growth factor receptor; PSA, Prostate-specific antigen; PCWG2, Prostate Cancer  Clinical Trials Working Group 2; RECIST, Response evaluation criteria in solid tumors;  VEGF, Vascular endothelial growth factor; VEGFR, Vascular endothelial growth factor  receptor; VHL, Von Hippel Lindau.

## Competing interests

CH declares that she has no competing interests. EH reports receiving research funding from Astra Zeneca, GlaxoSmithKline and Pfizer.

## Authors' contributions

CH drafted the manuscript. EH conceived of the manuscript and performed critical revisions. Both authors read and approved of the final manuscript.
